# Electrochemical cyclization of alkynes to construct five-membered nitrogen-heterocyclic rings

**DOI:** 10.3762/bjoc.21.166

**Published:** 2025-10-16

**Authors:** Lifen Peng, Ting Wang, Zhiwen Yuan, Bin Li, Zilong Tang, Xirong Liu, Hui Li, Guofang Jiang, Chunling Zeng, Henry N C Wong, Xiao-Shui Peng

**Affiliations:** 1 Key Laboratory of Theoretical Organic Chemistry and Functional Molecule of Ministry of Education, School of Chemistry and Chemical Engineering, Hunan University of Science and Technology, Xiangtan, Hunan 411201, P. R. China; 2 School of Science and Engineering, Shenzhen Key Laboratory of Innovative Drug Synthesis, The Chinese University of Hong Kong, Shenzhen, Shenzhen 518172, P. R. China; 3 Hunan Norchem Pharmaceutical Company, Ltd., Changsha 410000, P. R. China; 4 College of Chemistry and Chemical Engineering, Hunan University, Changsha 410082, P. R. China

**Keywords:** alkyne, catalysis, cyclization, electrochemistry, five-membered ring

## Abstract

Organic five-membered rings have shown significant applications in the fields of organic synthesis, natural products, organic materials and pharmaceuticals for their unique characteristics. Electrochemical construction of five-membered rings from alkynes attracted increasing attention due to the notable advantages of electrochemical transformations and facile access of alkynes. Indole skeletons were constructed successfully through electrochemical intramolecular coupling of ethynyl-involved ureas, annulation of *o*-arylalkynylanilines, cyclization of 2-ethynylanilines, selenocyclization of diselenides with 2-ethynylanilines as well as C–H indolization of 2-alkynylanilines with 3-functionalized indoles. Isoindolones were synthesized successfully by electrochemical annulation of benzamides with terminal alkynes, 5-*exo-dig* aza-cyclization of 2-alkynylbenzamides as well as reductive cascade annulation of *o*-alkynylbenzamides. Pyrroles and imidazoles were formed efficiently via electrochemical annulation of alkynes with enamides and tandem Michael addition/azidation/cyclization of alkynes, amines and azides, respectively. Imidazopyridines could be obtained by electrochemical [3 + 2] cyclization of heteroarylamines. The electrochemical oxidative [3 + 2] cycloaddition of secondary propargyl alcohols was a simple and efficient access towards 1,2,3-triazoles. In this review, electrochemical cyclizations of alkynes to construct five-membered rings are highlighted. Firstly, the property and application of five-membered rings are simply introduced. After presenting the usefulness of alkynes and the general progress of electrochemical transformations, electrochemical cyclization reactions of alkynes towards five-membered rings are classified and presented in detail. Based on different types of five-membered rings, electrochemical construction of indoles, isoindolinones, indolizines, oxazoles, imidazoles, pyrroles, imidazoles and 1,2,3-triazoles are summarized and the possible reaction mechanisms are disclosed if available.

## Introduction

**Figure 1:**
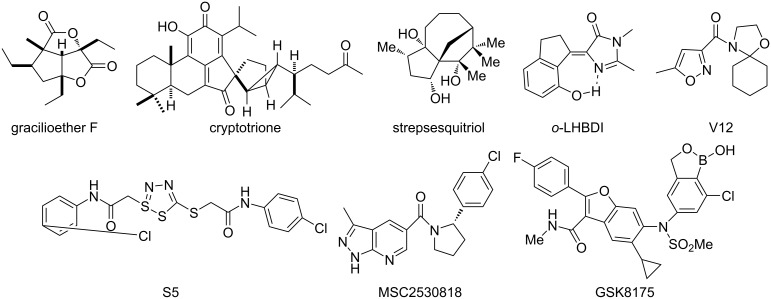
Natural products and functional molecules possessing five-membered rings.

Organic five-membered rings, an essential class of organic compounds, not only are frequently used as important starting materials, intermediates or ligands in organic synthesis [1–14] but also are critical moieties in natural products [15–18], organic materials [19] and pharmaceuticals [20–24] due to their unique chemical, electrical, optical, pharmacological and biological properties. Gracilioether F and cryptotrione have fused and spiro five-membered rings, respectively [25–26]. Strepsesquitriol bearing bridged five-membered rings was firstly synthesized by Li in 2024 [27]. The green fluorescent protein (GFP) core chromophore (*o*-LHBDI) displayed a potential application in organic light emitting diodes [28]. Formyl oxazolidine (V12) was a potential candidate to protect maize from herbicide harm [29]. Thiadiazole-linked thioacetamide (S5) exhibited exceptional inhibitory activity against *Synechocystis* sp. PCC6803 and Cy-FBP/SBPase [30]. Pyrrolidine compound MSC2530818 could be potentially used as an inhibitor of cyclin dependent kinase (CDK8) [31]. Sulfonamide-*N*-benzoxaborole analog GSK8175 is an inhibitor against hepatitis C virus (HCV) [20] (Figure 1).

The construction of five-membered rings obtained growing attention [32–38], and alkynes [39–55] have been extensively applied as facilely available starting materials to build five-membered rings for their hybrid structures with appropriate reactivities [56–59]. For example, cyclizations of silyloxyenyne [60], anionic cyclization of enediyne [61], [3 + 2] reductive cycloadditions of enal-alkyne [62], [2 + 2 + 1] cycloaddition of acetylenes [63] and cyclization of 1,6-enyne [64] were efficient approaches towards five-membered rings. Since Faraday synthesized hydrocarbons by employing electric current to an acetate solution [65], the use of electricity to promote a reaction grew up gradually [56–74]. In the past decades, the electrochemical organic reactions [75–88] which utilized an external applied voltage to accelerate transformations far from thermodynamic equilibria have emerged abundantly with the consideration of green chemistry [89–92]. Redox-active organic compounds, transition metal coordinating compounds and even an electrode surface were commonly employed as catalysts in the electrochemical transformations [93–94]. Electrochemical transformations used renewable and clean electricity as a source of electrons and electron holes to generate radical species, showing several superiorities such as safety, economy, high selectivity, scalability, mild reaction conditions, powerful efficiency, environment-friendly and sustainability [95–100]. Numerous electrochemical constructions of cyclic compounds from alkynes have been developed. For examples, 2-aryl-3-sulfonyl-functionalized quinoline was formed by an electrochemical annulation of benzoxazinanone and *p*-arylsulfonyl hydrazide [101]. The electro-oxidative annulation of alkyne and benzamide afforded chiral pyridine-*N*-oxide [102], isoquinoline was synthesized successfully via electrochemical annulation of alkyne and benzamide [103] or imidate [104], electrochemical annulation of alkyne and acrylamide afforded α-pyrone and α-pyridone [105], sultam-fused pyridinone [106] as well as cyclicphosphinic amide [107] were produced by electrochemical cyclization of alkyne. Especially, the electrochemical organic transformation of alkyne was widely applied to build five-membered rings. For example, benzimidazole-fused isoindole was generated by electrochemical [4 + 1] annulation of alkynoate with arylbenzimidazole [108], and the electrochemical *ortho*-annulation of 2-alkynylbenzenesulfonamide gave the corresponding five-membered heterocycle [106].

In recent years, a few reviews about the electrochemical cyclization of alkynes and electrochemical synthesis of cyclic compounds have emerged. The electrochemical functionalization of alkynes was highlighted by Ahmed in 2019 [109], Zhang described radical annulation of 1,*n*-enynes under photo/electrochemical reaction conditions in 2023 [110], the electrochemical formation of heterocycles was summarized by Sindhu in 2022 [98] and sustainable syntheses of heterocycles from alkyne annulations through C–H activations were reported by Ackermann in 2024 [111]. Although few examples about the electrochemical formation of five-membered rings from alkynes were included in the above reviews [106,111], a systemic review on electrochemical construction of five-membered rings from alkynes was in high need due to the importance of five-membered rings and the advantages of electrochemical transformations. In this review, we summarize the advance on electrochemical construction of five-membered rings from alkynes systematically. According to different types of five-membered rings, the electrochemical construction of five-membered rings from alkynes are mainly classified into the following categories: (a) construction of indoles, (b) construction of isoindolinones and indolizines, (c) construction of oxazoles and imidazoles, (d) construction of pyrroles, imidazoles and 1,2,3-triazoles. Literature on the electrochemical formation of five-membered rings from alkynes in this review was collected up to June 2025. We apologize to the authors if their contributions were not involved here due to the limitations of the search tools and profiles applied.

## Review

### Construction of indoles

**Scheme 1:**
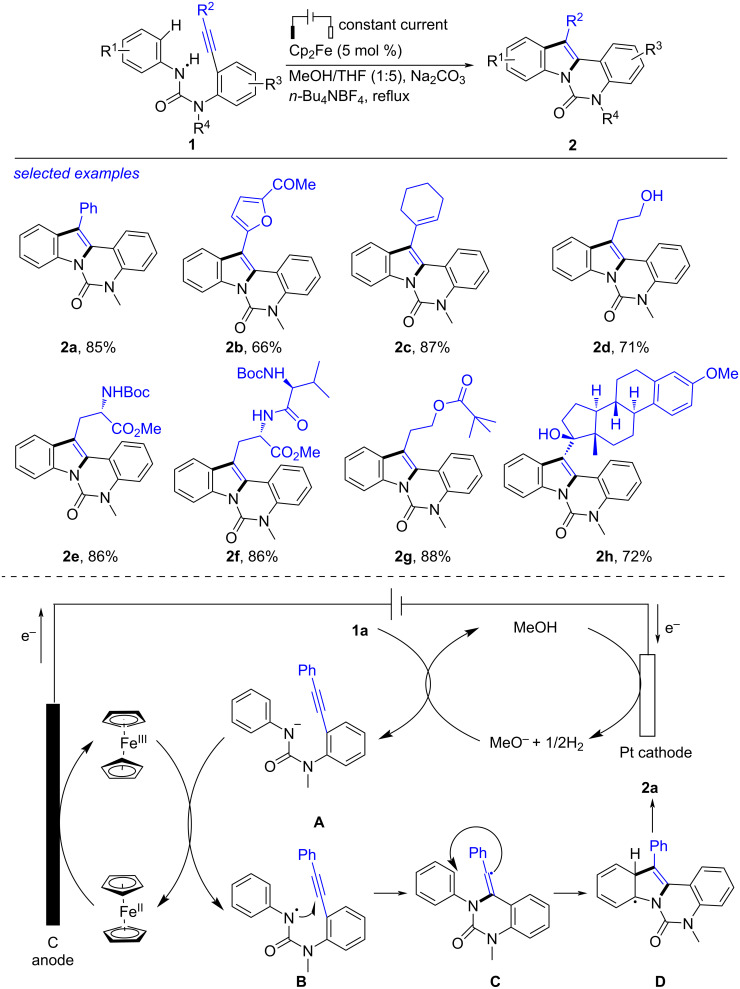
Electrochemical intramolecular coupling of ureas to form indoles.

Indoles, exhibiting interesting photoelectric properties and biological activities, were widely applied in organic synthesis, pharmacology and organic materials science [112–136]. Recently, Shi has disclosed the synthesis or transformation of indoles and developed indole-derived platform molecules [137–161]. Besides, electrochemical cyclization of alkynes is also an important access towards indoles. In 2016, Xu reported the electrochemical intramolecular coupling of urea derivatives to form substituted indoles (Scheme 1) [162]. Using [Cp_2_Fe] (5 mol %) as the redox catalyst, the intramolecular coupling of ureas **1** proceeded smoothly in an undivided cell (reticulated vitreous carbon (RVC) anode, Pt cathode, 5 mA), forming the desired indoles **2** in high yields. The reaction showed good compatibility with various functional groups like phenyl, furyl, alkenyl and alkyl at the acetylene moieties, producing **2a**–**d** in 66%–87% yields. Boc-amino ester (**2e**), dipeptide (**2f**), apivalate ester (**2g**) and ethinyl estradiol (**2h**) skeletons were also tolerated well. According to the previous works [163] and the experimental results, the authors proposed a plausible mechanism. Firstly, the anodic oxidation of [Cp_2_Fe] generated [Cp_2_Fe]^+^ along with cathodic reduction of MeOH to H_2_ and MeO^−^ acting as a base. Deprotonation of **1a** using MeO^−^ produced the anion **A**, which underwent single-electron transfer (SET) with [Cp_2_Fe]^+^ to give the nitrogen-centered radical **B** with regeneration of [Cp_2_Fe] [164–172]. Then, the 6-*exo-dig* cyclization of **B** obtained the vinyl radical **C** [173] that proceeded cyclization with the aryl species to form the radical **D**. Eventually, the rearomatization of **D** by eliminating one proton along with electron afforded **2a**. This protocol, proceeding smoothly without noble-metal catalyst and oxidant, was an economic and efficient protocol compared with the previous method [174–184].

**Scheme 2:**
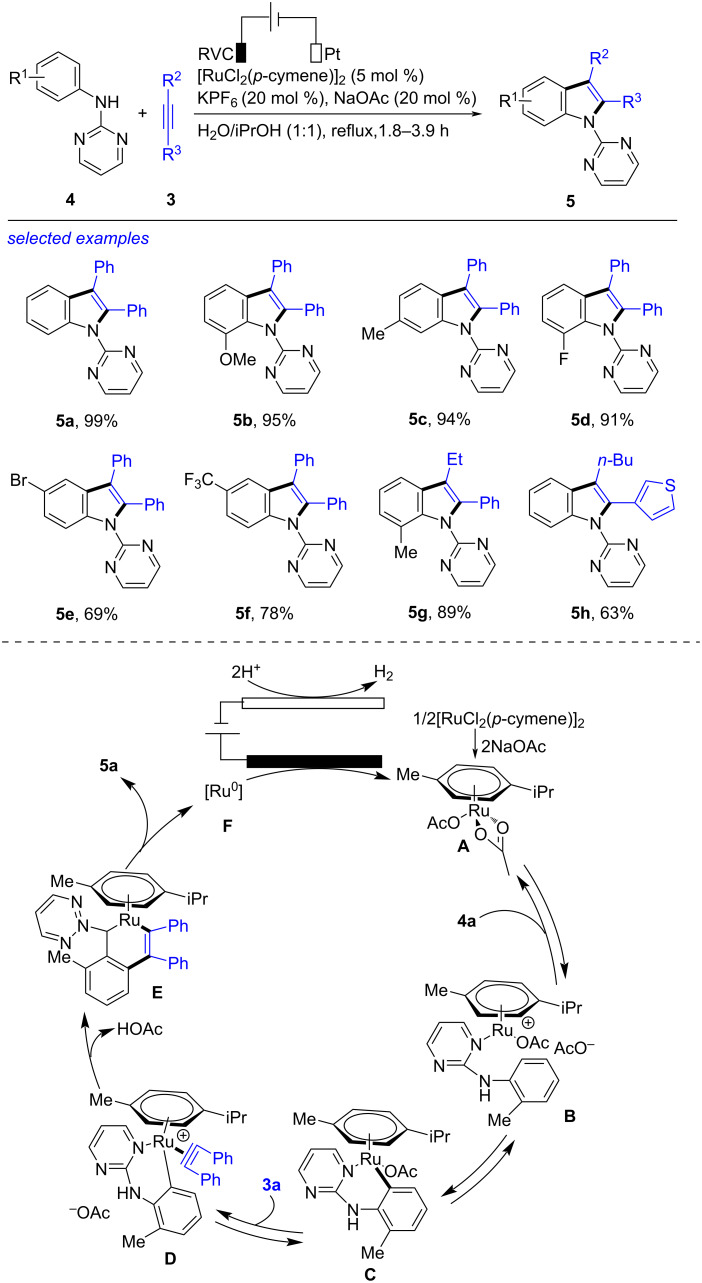
Electrochemical dehydrogenative annulation of alkynes with anilines.

The ruthenium-accelerated electrochemical dehydrogenative annulation of alkyne with an aniline derivative was also an efficient method to build the indole frame (Scheme 2) [185]. In the presence of KPF_6_ and NaOAc, subjection of alkyne **3** and aniline **4** to [RuCl_2_(*p*-cyneme)]_2_-catalyzed electrochemical annulation formed the titled indole **5** successfully. After studying the reaction in details, the best reaction conditions were acquired as following: a mixture of aniline **4** (0.3 mmol), alkyne **3** (0.6 mmol), [RuCl_2_(*p*-cymene)]_2_ (0.03 mmol), KPF_6_ (0.06 mmol) and NaOAc (0.06 mmol) in H_2_O/iPrOH (1:1, 6 mL), refluxing under electrolysis (RVC anode, Pt cathode, 10 mA) for 1.8–3.9 h. This reaction was compatible with anilines with either electron-donating (MeO, Me) or electron-withdrawing (F, Br, CF_3_) groups on the phenyl cycle to generate the corresponding products **5b**–**f** in moderate to excellent yields. The internal alkynes incorporated with phenyl and ethyl, butyl and thienyl were applicable in this transformation, leading to the formation of **5g** and **5h** in 89% and 63% yield, respectively. Based on the results of control experiments and the previous reports [186], a plausible reaction mechanism was deduced. Firstly, treatment of [RuCl_2_(*p*-cymene)]_2_ with NaOAc afforded the ruthenium diacetate species **A**, which underwent complexation with **4** and reversible C–H activation to give the six-membered intermediate **C**. Substitution of the acetate ligand in **C** by **3** caused the generation of complex **D**. The six-membered ruthenacycle **E** was then obtained by migratory insertion of acetylene into the Ru–C bond. Finally, reductive elimination of **E** formed the target indole **5** and a Ru(0) species **F** that was oxidized on the RVC anode to regenerate **A**. This electrochemical formation of indole, using easily available reactants and proceeding successfully under aqueous solution with simple undivided cell, was a green and convenient route towards indole.

**Scheme 3:**
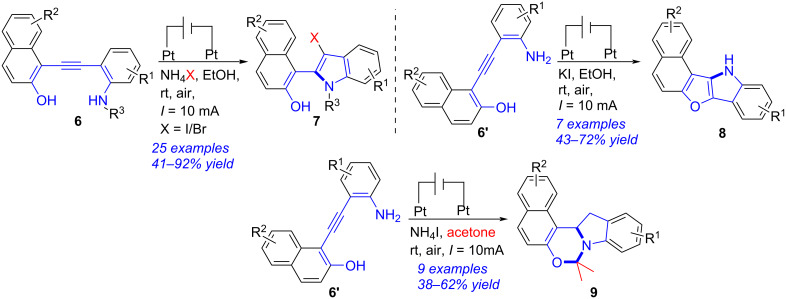
Electrochemical annulations of *o*-arylalkynylanilines.

A series of skeletally diverse indoles were obtained successfully via electrochemical annulations of *o*-arylalkynylanilines (Scheme 3) [187]. In an undivided cell (Pt anode, Pt cathode, 10 mA), treatment of *o*-arylalkynylanilines **6** with ammonium halides (NH_4_X, X = I, Br, Cl) gave the C3-halogenated indoles **7** in moderate to excellent yields. When KI was used instead of NH_4_X, naphtho[1′,2′:4,5]furo[3,2-*b*]indoles **8** were generated in 43–72% yields. Performing the electrochemical bicyclization of **6'** with NH_4_I in acetone yielded naphtho[1′,2′:5,6][1,3]oxazino[3,4-*a*]indoles **9** in moderate yields. It was worth mentioning that this report provided a switchable and green synthetic methodology for skeletally diverse indoles.

**Scheme 4:**
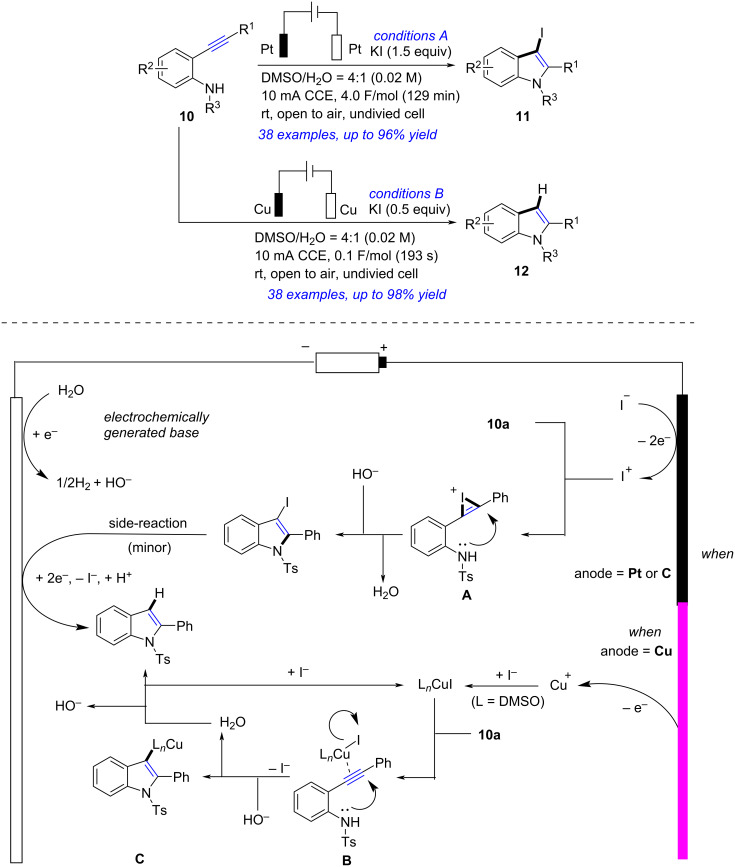
Electrochemical cyclization of 2-ethynylanilines.

Divergent electrochemical cyclization of 2-ethynylanilines was developed to synthesize indoles and iodoindoles (Scheme 4) [188]. Treatment of 2-ethynylanilines **10** with KI in DMSO/H_2_O in an undivided cell (Pt electrodes, 10 mA, 4.0 F/mol) afforded 3-iodoindoles **11** in satisfactory yields. When an alternative cell (Cu electrodes, 10 mA, 0.1 F/mol) was applied, the target indoles **12** were obtained in excellent yields. On the basis of control experiments and previous studies [189–200], the authors proposed a possible reaction mechanism. For the synthesis of **11a** in Pt plate electrodes, two-electron anodic oxidation of I^−^ formed I^+^. Addition of I^+^ to C≡C in **10** resulted in the production of **A**. Meanwhile, continuous reduction of H_2_O at the cathode formed H_2_ and HO^−^. The *anti-*nucleophilic attack of the N atom in **A** and the following HO^−^ facilitated deprotonation and formed the corresponding 3-iodoindole **11a**. Excessive-reduction (a minor side-reaction) of **11a** took place as well in certain instances, resulting in the formation of **12a**. And for the generation of **12a** in Cu rod electrodes, the Cu anode was expected to liberate Cu^+^ into the reaction mixture. The reaction of this Cu^+^ with DMSO and I^−^ afforded (DMSO)*_n_*CuI, which was coordinated with C≡C to give **B**. The intermediate **C** was obtained by cyclization of **B** and deprotonation. Further protonation of **C** afforded **12a** and regenerated (DMSO)*_n_*CuI. Notably, this reaction, using KI as the only additive and performing under ambient conditions in a non-volatile aqueous solvent, was a simple, selective, efficient and sustainable electrosynthesis of indole derivatives.

**Scheme 5:**
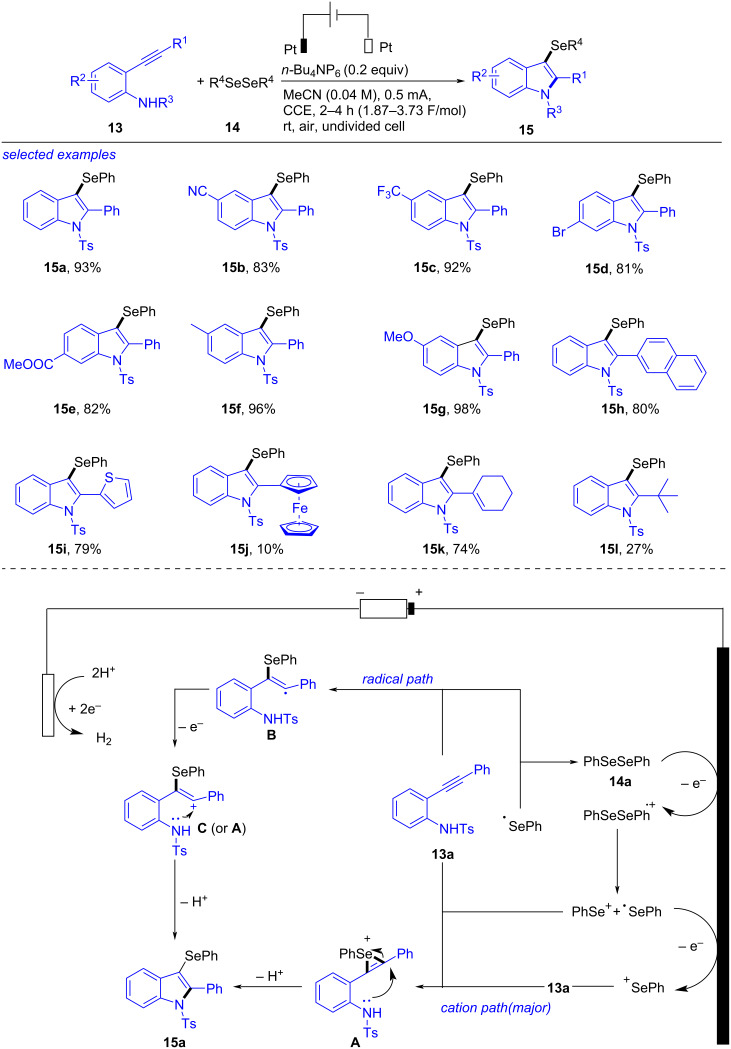
Electrochemical selenocyclization of diselenides and 2-ethynylanilines.

3-Selenylindoles were also formed by electrochemical selenocyclization of diselenides and 2-ethynylanilines (Scheme 5) [201]. After probing the reaction systematically, the optimal conditions were afforded as following: a mixture of 2-ethynylaniline **13** (0.2 mmol), diselenide **14** (0.13 mmol), *n-*Bu_4_NPF_6_ (0.04 mmol) and MeCN (5.0 mL), under electrolysis (Pt plate electrodes, 5 mA, 1.87 F/mol) at rt for 2–4 h. 2-Ethynylanilines with either electron-withdrawing (CN, CF_3_, Br, COOMe) or electron-donating (Me, OMe) groups at the phenyl cycle of aniline were tolerated well under these conditions, producing the corresponding **15b**–**g** in 81–98% yield. This reaction also showed high compatibility with 2-naphthyl (**15h**), 2-thiophenyl (**15i**), ferrocenyl (**15j**), cyclohexenyl (**15k**) and *tert-*butyl (**15l**) incorporated at the ethynyl moiety. According to the results of control experiments, a plausible mechanism was presented. Firstly, one-electron oxidation of **14a** occurred to give a radical cation PhSeSePh^•+^ at the anode. The subsequent cleavage of Se–Se bond formed a radical PhSe^•^ and a cation PhSe^+^. Further additional oxidation of PhSe^•^ yielded another PhSe^+^, which worked as the major reactive species and quickly added to C≡C in **13a** to form intermediate **A**. Finally, **A** proceeded an intramolecular nucleophilic attack by N and deprotonation to finish **15a**. The other possible pathway was radical route, in which PhSe^•^ dimerized to reform **14a** or added to C≡C bond in **13a** to afford **B**. The subsequent anodic oxidation of **B** gave **C**, which underwent nucleophilic cyclization/deprotonation to form the target **15a**. Meanwhile, H^+^ was continually reduced at the cathode to give by-product H_2_. This transformation, completing under short reaction time and conditions with a low equivalent of charges with high yields and good substrate scope, was a convenient, efficient, practical and a sustainable strategy for the preparation of 3-selenylindoles.

**Scheme 6:**
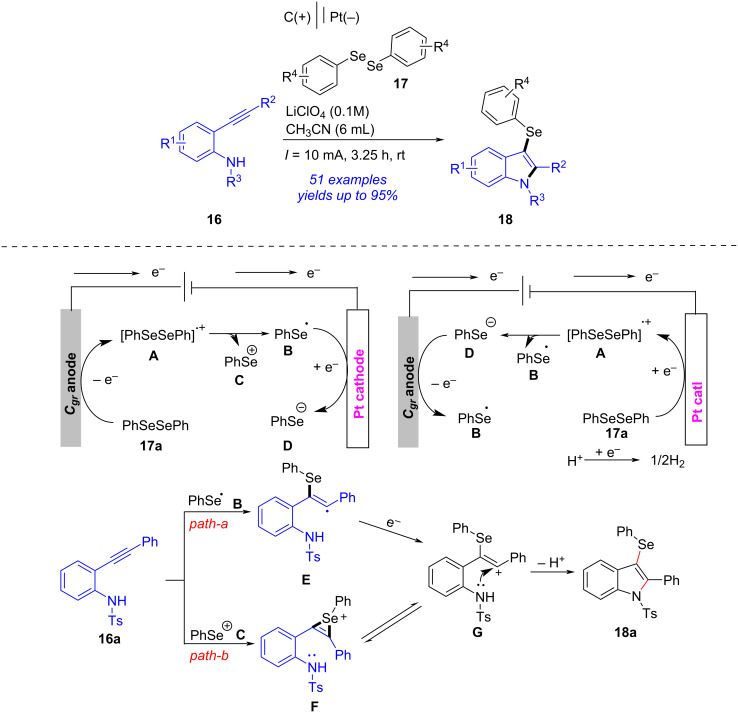
Electrochemical cascade approach towards 3-selenylindoles.

In 2023, Satyanarayana also described a similar electrochemical cascade approach towards 3-selenylindoles from 2-alkynylanilines (Scheme 6) [202]. When graphite was used as anode, platinum as cathode and LiClO_4_ as electrolyte, the electrochemical oxidative cyclization of 2-alkynylaniline **16** and diselenide **17** occurred to form desired 3-selenylindole **18** in satisfactory yields with wide substance scope. Based on control experiments and previous references [203], a possible reaction mechanism was outlined. Firstly, the anodic oxidation of **17a** formed phenylselenium cation **C** and phenylselenium radical **B** through radical cation species **A**. Simultaneously, the cathodic reduction of **17a** generated anion **D** and radical **B**. Then, addition of **B** with the alkyne portion in **16a** gave a radical intermediate **E**, which proceeded a one-electron oxidation followed by nucleophilic addition and then deprotonation to yield the desired **18a** via intermediate **G**. Another possible pathway is that phenylselenium cation **C** attacked **16a** afforded the alkenyl cation **G**, which underwent cyclization and deprotonation to produce **18a**. It should be noted that this conversion proceeded under metal, oxidant, and base-free conditions.

**Scheme 7:**
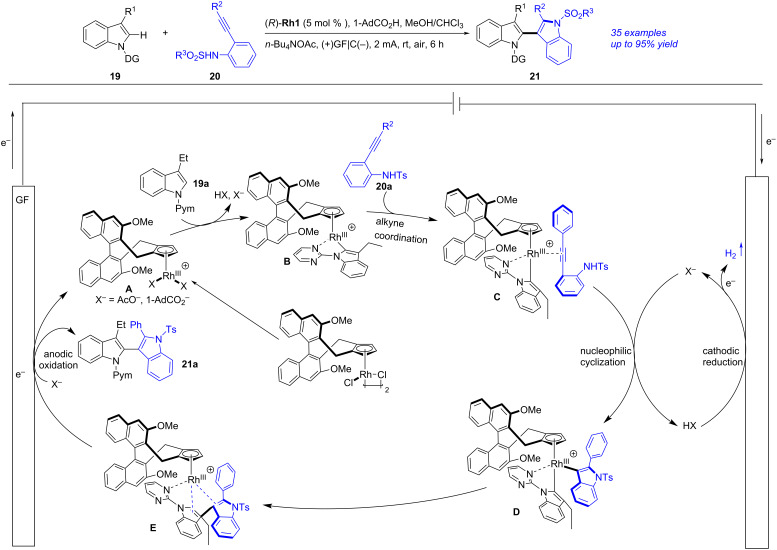
Electrochemical C–H indolization.

An electrochemical enantioselective tandem C–H indolization of 2-alkynylanilines with 3-functionalized indoles towards 2,3′-biindolyl atropisomers was achieved by Zeng in 2025 (Scheme 7) [204]. After screening the reaction carefully, the optimal conditions were gained as following: a mixture 3-functionalized indole **19** (0.08 mmol), 2-alkynylaniline **20** (0.12 mmol), (*R*)-**Rh1** (5 mol %), *n-*Bu_4_NOAc (0.08 mmol), 1-adamantane carboxylic acid (1-AdCO_2_H, 0.08 mmol) and MeOH/CHCl_3_ (1:1, 2 mL), under electrolysis (graphite felt (GF) anode and graphite (C) cathode, 2 mA, 5.6 F/mol) at rt for 6 h. The authors also proposed the reaction mechanism on basis of experimental results and previous literature [205–206]. Firstly, the ligand exchange between (*R*)-**Rh1** and *n-*Bu_4_NOAc or 1-AdCO_2_H gave a chiral active catalyst **A**. The irreversible base-prompted C–H activation of **A** with **19a** yielded a five-membered rhodacycle **B**, which underwent alkyne coordination followed by nucleophilic cyclization with **20a** to give the biindolyl–Rh species **D**. The reductive elimination of **D** produced the bisindole-ligated CpxRhI intermediate **E**, which performed anodic oxidation to finish **21a** and regenerate **A**. Notably, this protocol was an efficient and sustainable approach to synthesize 2,3′-biindolyl atropisomers and could be potentially applied in manufacture of functional materials, bioactive molecules and chiral ligands.

### Construction of isoindolinones and indolizines

**Scheme 8:**
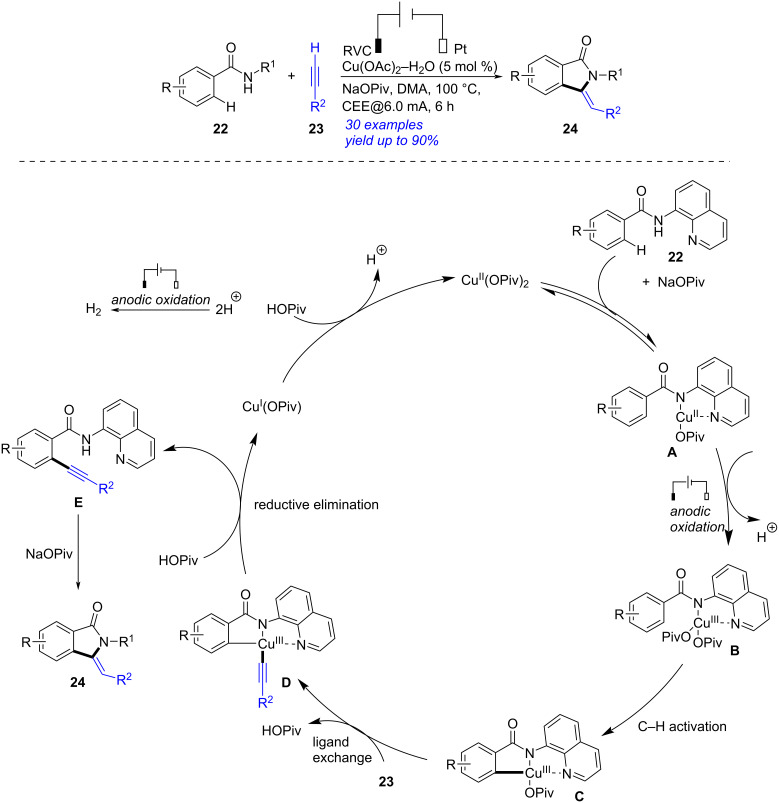
Electrochemical annulation of benzamides and terminal alkynes.

An electrochemical and copper-catalyzed annulation of benzamides and terminal alkynes was established for the synthesis of isoindolones by Ackermann in 2019 (Scheme 8) [207]. After screening the reaction carefully, the optimum conditions were presented as following: a mixture of benzamide **22** (0.25 mmol), alkyne **23** (0.50 mmol), NaOPiv (0.25 mmol), Cu(OAc)_2_-H_2_O (0.0125 mmol) and *N*,*N*-dimethylacetamide (DMA, 4.0 mL), under electrolysis (RVC anode, Pt cathode, 6.0 mA) at 100 °C for 6 h. According to the experimental results, a proposed mechanism was outlined. Firstly, treatment of NaOPiv (0.25 mmol) with Cu(OAc)_2_-H_2_O formed Cu(OPiv)_2_. Coordination of **22** with Cu(OPiv)_2_ and the following anodic copper(II) oxidation provided copper(III) carboxylate intermediate **B**. Facile carboxylate-promoted C–H activation and ligand exchange with **23** formed the copper(III) species **D**, which underwent metalation/reductive elimination to generate intermediate **E** along with the formation of Cu(OPiv) which was transformed to Cu(OPiv)_2_ by oxidation at the anode. Finally, the cyclization of **E** afforded target isoindolone **24**. Notably, this reaction was the first example of electrochemical copper-catalyzed oxidative cyclization of alkyne which was enabled by C–H alkynylation of electron-deficient benzamide.

**Scheme 9:**
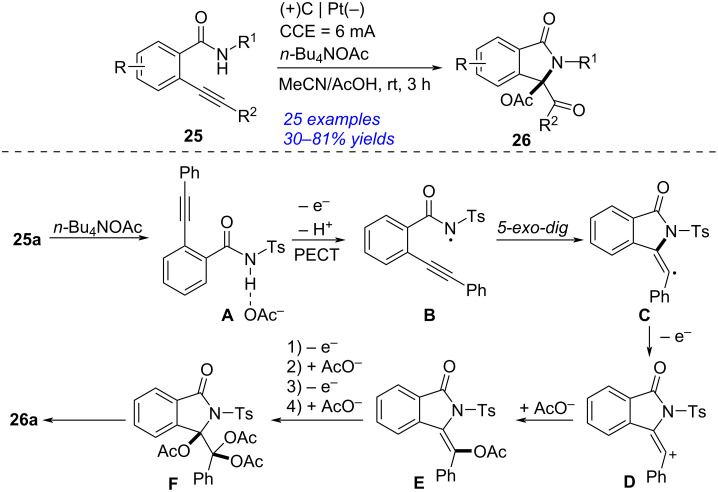
Electrochemical synthesis of isoindolinone by 5-*exo-dig* aza-cyclization.

In 2022, Ye presented an electrochemical synthesis of isoindolinone through 5-*exo-dig* aza-cyclization of 2-alkynylbenzamide (Scheme 9) [208]. By applying carbon cloth as anode, Pt as cathode and *n-*Bu_4_NOAc as electrolyte, the 5-*exo-dig*/6-*endo-dig* cyclization of 2-alkynylbenzamide **25** occurred to form the corresponding isoindolinone **26** in reasonable yields. According to the experimental results and previous investigations [209–211], the proposed reaction mechanism was described. Initially, proton-coupled electron transfer took place between *n-*Bu_4_NOAc and 2-(phenylethynyl)-*N*-tosylbenzamide (**25a**) to afford the amidyl radical **B**, which then proceeded intramolecular 5-*exo-dig* radical annulation to form the five-membered intermediate **C**. The oxidation of **C** followed by capturing an AcO^−^ generated the intermediate **E**, which was converted into triacetate adduct **F** through anodic oxidation and AcO^−^ capture. The hydrolysis of **F** then occurred to afford the final product **26a**. This protocol featured with some advantages such as without any oxidants and metal catalysts, simple operation, good yields, high selectivity and wide substrate scope.

**Scheme 10:**
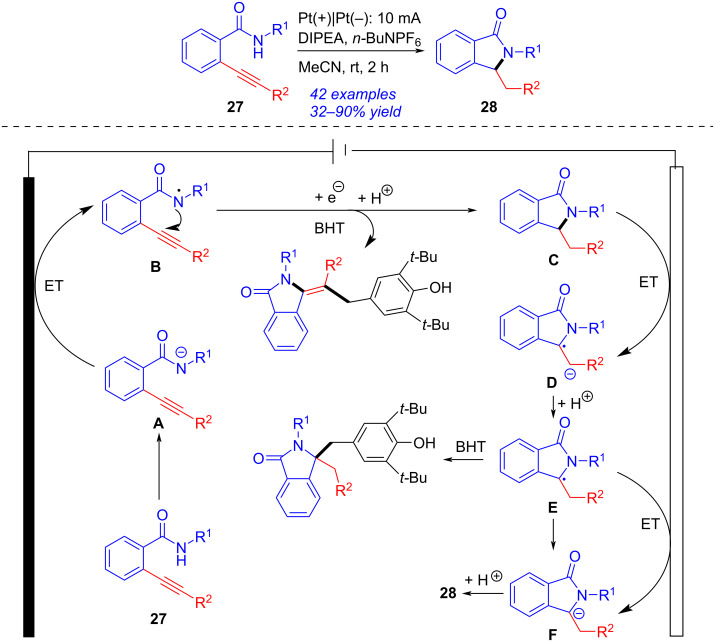
Electrochemical reductive cascade annulation of *o*-alkynylbenzamide.

Isoindolinone could be also obtained by electrochemical reductive cascade annulation of *o*-alkynylbenzamide (Scheme 10) [212]. Under 10 mA constant current with two platinum plate as electrodes and *n-*Bu_4_NPF_6_ as electrolyte, the reductive cascade cyclization of *o*-alkynylbenzamide **27** proceeded smoothly in the presence of *N*,*N*-diisopropylethylamine (DIPEA) to give the corresponding isoindolinone **28** in 32–90% yield. A plausible reaction mechanism was presented according to the experimental results and earlier works [213–214]. Firstly, the proton coupled electron transfer (PCET) procedure of **27** formed the amidyl radical **B**, which performed 5-*exo-dig N*-radical addition into the C≡C bond to generate the cyclic species **C**. The radical anion **D** was then obtained via single electron reduction of **C** at the cathode. The subsequent protonation of **D** gave α-aminyl radical **E** [215–217], which was converted into the anion **F** by further cathodic reduction. The subsequent protonation of **F** occurred to complete the formation of **28**. This approach, applying electrolyte as the proton sources, avoided the use of reductants and metal catalysts efficiently.

**Scheme 11:**
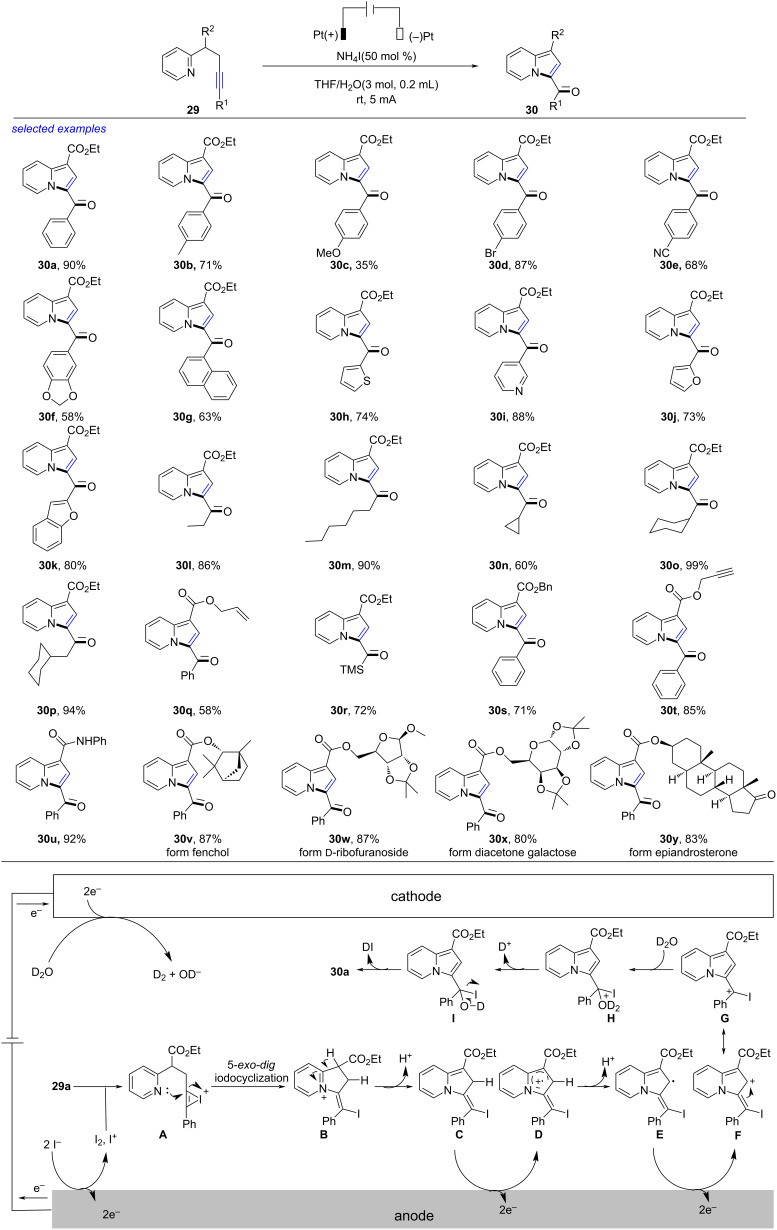
Electrochemical intramolecular 1,2-amino oxygenation of alkyne.

In 2022, Guo developed an electrochemical intramolecular 1,2-amino oxygenation of alkyne to access indolizine (Scheme 11) [218]. Under electrolysis (two platinum plate as electrodes, NH_4_I as electrolyte and electrocatalyst, 5 mA, 8.4 F/mol), the aminooxygenation of alkyne **29** underwent efficiently to form the desired indolizine **30** in good to excellent yield. The reaction was compatible with many groups involved at the ethyne moiety like substituted phenyl (**30b**–**e**), benzodioxole (**30f**), naphthyl (**30g**), thienyl (**30h**), pyridinyl (**30i**), furyl (**30j**), benzofuranyl (**30k**), alkyl (**30l**, **30m**), cycloalkyl (**30n**–**p**) and trimethylsilyl (**30r**). This reaction was also compatible with benzyl ester (**30s**), propynyl ester (**30t**), phenylamide (**30u**) as well as natural product-derived and pharmaceutical skeletons: fenchol (**30v**), ᴅ-ribofuranoside (**30w**), diacetone galactose (**30x**), and epiandrosterone (**30y**). Based on the experimental results, a possible reaction mechanism was disclosed. Initially, the anodic oxidation of I^−^ formed I^+^ or I_2_. Coordination of **29a** with I^−^ produced the iodonium species **A**, which was transformed to vinyl iodide **B** by intramolecular 5-*exo-dig* iodocyclization. The deprotonation/anodic oxidation of **B** gave the radical cation **D** via **C**. The second deprotonation/anodic oxidation produced **F**, which was transformed into stable cationic resonance **G** quickly. The nucleophilic attack of D_2_O formed **H**, which underwent dedeuteration and elimination of DI to form **30a** with reduction of deuterated water to generate deuteroxyl ions (OD^−^) and deuterium gas (D_2_) at the cathode. This method, using iodide salts as electrolyte and redox mediator and proceeding in aqueous solution with pleasure yields, was a simple, convenient, powerful, environmentally benign and sustainable electrooxidative approach towards indolizine.

### Construction of oxazoles and imidazoles

**Scheme 12:**
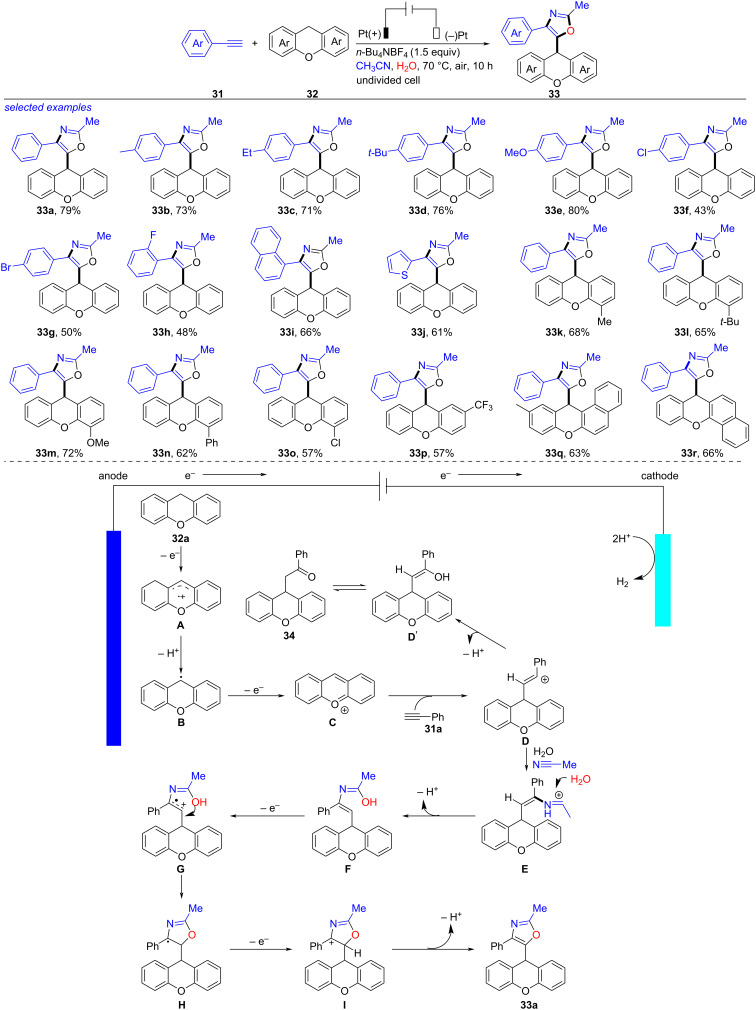
Electrochemical multicomponent reaction of nitrile, (thio)xanthene, terminal alkyne and water.

Without any additional oxidants and catalysts, an electrochemical multicomponent reaction of nitrile, xanthene, terminal alkyne and water to synthesize oxazole was established by Li in 2023 (Scheme 12) [219]. After examining the reaction carefully, the optimized reaction conditions were obtained as follows: a mixture of alkyne **31** (0.3 mmol), xanthene **32** (0.45 mmol), CH_3_CN (5.0 mL), H_2_O (0.3 mmol) and *n-*Bu_4_NBF_4_ (0.45 mmol), under electrolysis (Pt plate as electrodes, 5 mA) at air for 10 h. Electron-donating (Me, Et, *t-*Bu and OMe) or electron-withdrawing (Cl, Br and F) groups involved phenylacetylenes, 1-ethynylnaphthalene and 2-ethynylthiophene were tolerated well under these reaction conditions, resulting in the formation of the corresponding oxazoles (**33b**–**j**) in 43–80% yields. Xanthenes bearing Me, *t-*Bu, MeO, Ph, Cl, CF_3_ and naphthyl groups were applicable as well, generating the desired **33k**–**r** in moderate yields. According to the results of control experiments and previous studies [220–231], a proposed mechanism for this reaction was depicted. Firstly, the anodic oxidation of **32a** took place to give a radical cation species **A** that proceeded deprotonation to give benzylic radical intermediate **B**. Further oxidation of **B** afforded a cationic intermediate **C**, which was converted into **D** through nucleophilic addition of **31a**. Trapping **D** by the weak nucleophile H_2_O formed the by-product **34**, while trapping of **D** by CH_3_CN generated species **E**, which was trapped by H_2_O and formed intermediate **F**. Furthermore, oxidation of **F** at the anode produced radical cation **G** [232]. The following intramolecular cyclization/anode oxidation produced intermediate **I** that was then deprotonated to yield the target **33a**. Compared to the previous reported methods [233–254], this approach exhibited the following advantages like without metal catalysts and external oxidants, atom economy, facile access of starting materials, etc.

**Scheme 13:**
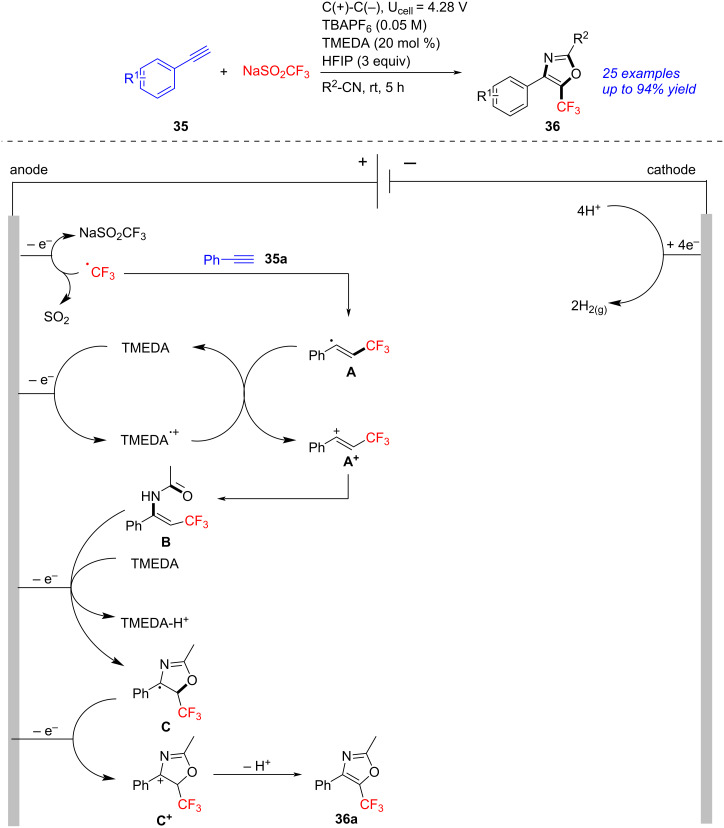
Electrochemical aminotrifluoromethylation/cyclization of alkynes.

In 2024, Cho succeeded in the preparation of trifluoromethylated oxazoles through in-situ aminotrifluoromethylation/cyclization of alkynes (Scheme 13) [255]. Under electrolysis (graphite as electrodes, tetra-*n-*butylammonium salt (TBAPF_6_) as electrolyte, *N*,*N*,*N*,*N*-tetramethylethylenediamine (TMEDA) as mediator, 4.28 V), the four-component reaction of alkynes **35**, NaSO_2_CF_3_, nitriles and residual water proceeded efficiently to form titled trifluoromethylated oxazoles **36** in moderate to excellent yields. A proposed mechanism was established on basis of control experiments. Firstly, the anodic oxidation of NaSO_2_CF_3_ gave a trifluoromethyl radical, which was then added to **35a**, affording alkenyl intermediate **A**. In the presence of TMEDA, oxidation of **A** yielded **A**^+^, which was then trapped by MeCN/H_2_O to form the CF_3_-enamide intermediate **B**. The subsequent cyclization/oxidation of **B** offered oxazoline radical intermediate **C**, which was transformed to the target **36a** through anodic oxidation/deprotonation. This transformation, implementing under mild conditions, was an efficient and straightforward protocol towards oxazoles.

**Scheme 14:**
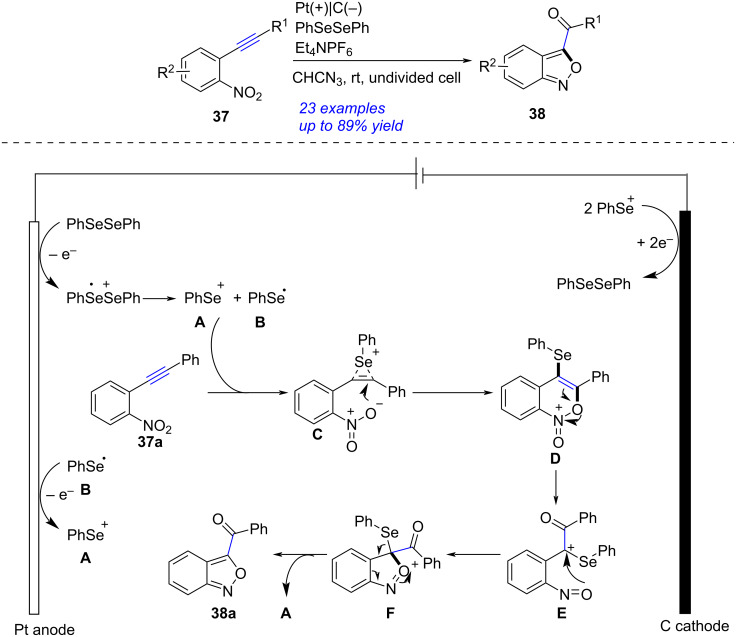
Electrochemical cyclization of *o*-nitrophenylacetylene.

An electrochemical and selenium-catalyzed construction of 2,1-benzoxazole through cyclization of *o*-nitrophenylacetylene was achieved by Pan in 2021 (Scheme 14) [256]. After examining the reaction in details, the best conditions were disclosed as following: a mixture of *o*-nitrophenylacetylene **37** (0.3 mmol), diphenyl diselenide (0.03 mmol), Et_4_NPF_6_ (0.15 mmol) and CH_3_CN (10 mL), under electrolysis (graphite cathode and platinum anode, 1.6 V) at rt. Based on the experimental results and the reported studies [257–258], the authors deduced a plausible reaction mechanism. Initially, the anodization of diphenyl diselenide produced phenylselenium radical **A** and selenium cation **B**, the single-electron transfer on the anode could also transformed **A** into **B**. Addition of **B** to **37a** formed the intermediate **C** that underwent intramolecular nucleophilic cyclization to give **D**. The fracture of the N–O bond in **D** yielded **E**. Elimination of selenium cation **A** from **E** and the following cyclization afforded **38a**. This transformation, combining selenium catalysis and organic electrosynthesis, proceeded smoothly at rt without external oxidants and metal catalysts.

### Construction of pyrroles, imidazoles and 1,2,3-triazoles

**Scheme 15:**
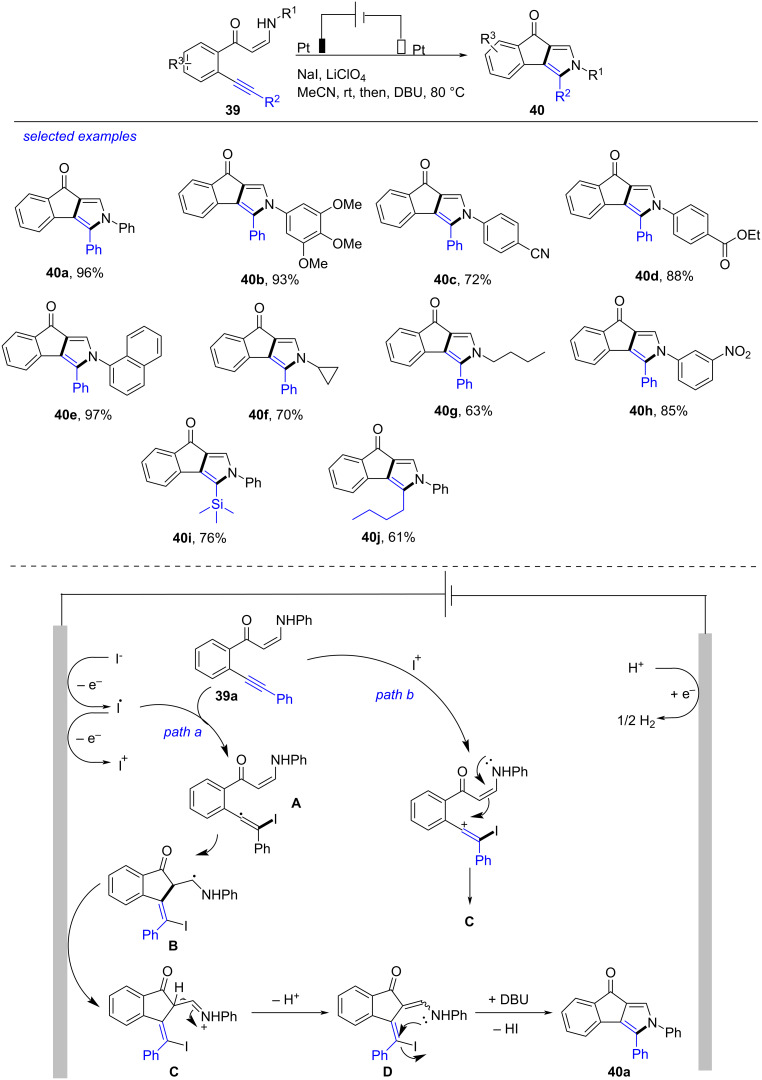
Electrochemical annulation of alkynyl enaminones.

A series of indeno[1,2-*c*]pyrroles were synthesized successfully through electrochemical annulation of alkynyl enaminones by Zhao in 2022 (Scheme 15) [259]. After examining the reaction carefully, the best reaction conditions were obtained as following: alkynyl enaminone **39** (0.2 mmol), LiClO_4_ (0.3 M) and NaI (0.1 M) in MeCN (6 mL) at 80 °C under electrolysis (Pt plate as electrodes, 10 mA) at rt for 20 h. Alkynyl enaminones bearing substituted phenyl, naphthyl, cyclopropyl and *n*-butyl groups at the amino moiety were tolerated well under these conditions, resulting in the formation of indeno[1,2-*c*]pyrroles **40b**–**g** in 63–97% yields. Alkynyl enaminones containing phenyl, trimethylsilyl and *n-*butyl groups at the ethynyl terminal were also applicable to produce the desired **40h**–**j** in satisfactory yields. Based on the results of control experiments and previous works [260–265], a proposed mechanism was also presented. Initially, I^−^ was oxidized to I^•^ at the anode. A vinyl radical intermediate **A** was produced through reaction of I^•^ with **39a**. The following intramolecular cyclization formed the species **B**, which was oxidized at the anode to generate imine intermediate **C**. Simultaneously, treatment of **39a** with I^+^ and the following intramolecular nucleophilic cyclization produced **C**. The elimination of a proton from **C** afforded the intermediate **D**. In the presence of 1,8-diazabicyclo[5.4.0]undec-7-ene (DBU), the intramolecular nucleophilic substitution of **D** afforded target indeno[1,2-*c*]pyrrole **40a** along with eliminating I. Notably, this oxidant-free and catalyst-free approach could be potentially applied in the pharmaceutical manufacture.

**Scheme 16:**
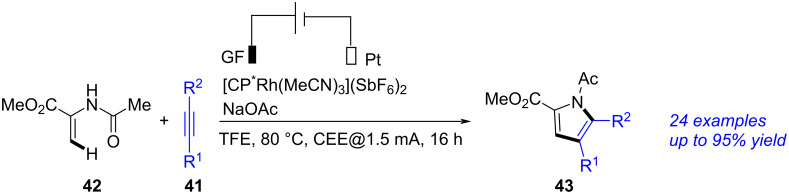
Electrochemical annulation of alkyne and enamide.

A Rh-promoted synthesis of pyrroles through annulation of alkynes and enamides was demonstrated by Ackermann in 2023 (Scheme 16) [266]. Using GF as anode, Pt as cathode, NaOAc as the electrolyte and [Cp*Rh(MeCN)_3_](SbF_6_)_2_ as the catalyst, the annulation between alkyne **41** and enamide **42** succeeded, forming the corresponding pyrrole **43** in good yield. Although this transformation employing electricity as a sustainable and green oxidant, noble metal catalyst (Rh) was required to achieve reasonable yields.

**Scheme 17:**
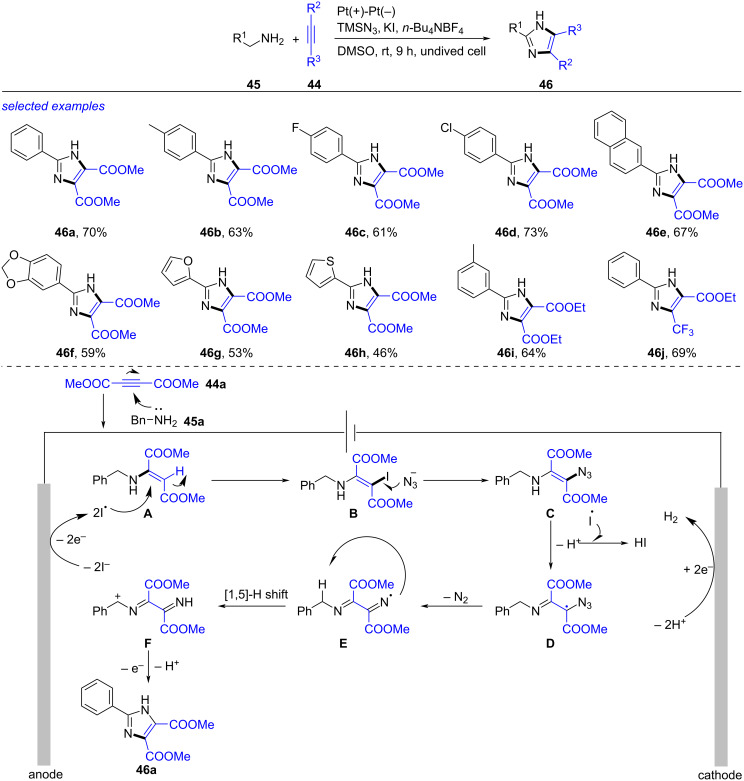
Electrochemical tandem Michael addition/azidation/cyclization.

An electrochemical synthesis of imidazoles through tandem Michael addition/azidation/intramolecular cyclization of alkynes, amines and azides was realized by Chen in 2022 (Scheme 17) [267]. After investigating the reaction in details, the optimum reaction conditions were acquired as following: a mixture of alkyne **44** (0.5 mmol), amine **45** (0.5 mmol), azidotrimethylsilane (TMSN_3_, 1.5 mmol), *n-*Bu_4_NBF_4_ (0.1 M), KI (1 mmol) and DMSO (5.0 mL), under electrolysis (Pt plate as electrodes, 9 mA) at rt for 9 h. The reaction was compatible with numerous amines such as substituted benzylamines **46b**–**d**, 1-(2-naphthyl)methanamine **46e**, 3,4-methylenedioxybenzylamine **46f**, furfurylamine **46g** and 2-thiophenemethylamine **46h**. Alkynes with ester and trifluoromethyl groups worked well under this reaction, leading to the corresponding imidazoles **46i**–**k** in moderate yields. With the consideration of experimental results and the reported literature [268–269], a proposed reaction mechanism was disclosed. Firstly, treatment of **44a** with **45a** formed **A**. Oxidation of I^−^ produced I^•^ that reacted with **A** to generate iodide **B**. Attachment of **B** by N_3_^−^ gave intermediate **C**. A free radical species **E** was obtained from **C** through oxidation and elimination of N_2_ via intermediate **D**. The subsequent [1,5]-H shift generated α-amino radical **F**, which was converted into the final product **46a** by oxidation and cyclization. This reaction avoided the use of metal catalysts and oxidants, but the yields remained to improve.

**Scheme 18:**
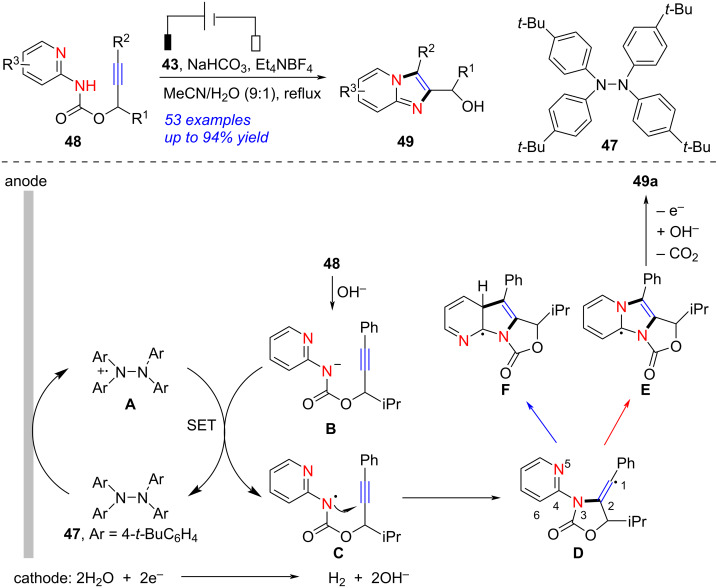
Electrochemical [3 + 2] cyclization of heteroarylamines.

An electrochemical [3 + 2] cyclization of heteroarylamine to access imidazopyridine was achieved by Xu in 2017 (Scheme 18) [270]. When RVC was used as anode, platinum as cathode and Et_4_NBF_4_ as electrolyte, the tetraarylhydrazine-(**47**)-catalyzed cyclization of heteroarylamine **48** succeeded, forming the corresponding imidazopyridine **49** in up to 94% yield with broad substance scope. The authors also presented a possible mechanism for this transformation. Firstly, the anodic oxidation of **47** formed a radical-cation species **A** along with the generation of OH^−^ through reduction of H_2_O at the cathode. In the presence of OH^−^, deprotonation of **48** underwent smoothly to generate the anion species **B**. The single-electron transfer from **B** to **A** gave amidyl radical **C** with the regeneration of **47**. The 5-*exo-dig* annulation of **C** provided vinyl radical intermediate **D**. The radical in **D** reacted with the pyridyl N atom regioselectively to form a tricyclic radical **E**, which proceeded one-electron oxidation/hydrolysis afforded **49**. Additionally, the imidazopyridines could be constructed through electrochemical intramolecular [3 + 2] annulation of carbamates as well [271]. Notably, the above approach provided imidazopyridines in high yields under aqueous solution without any metal catalysts.

**Scheme 19:**
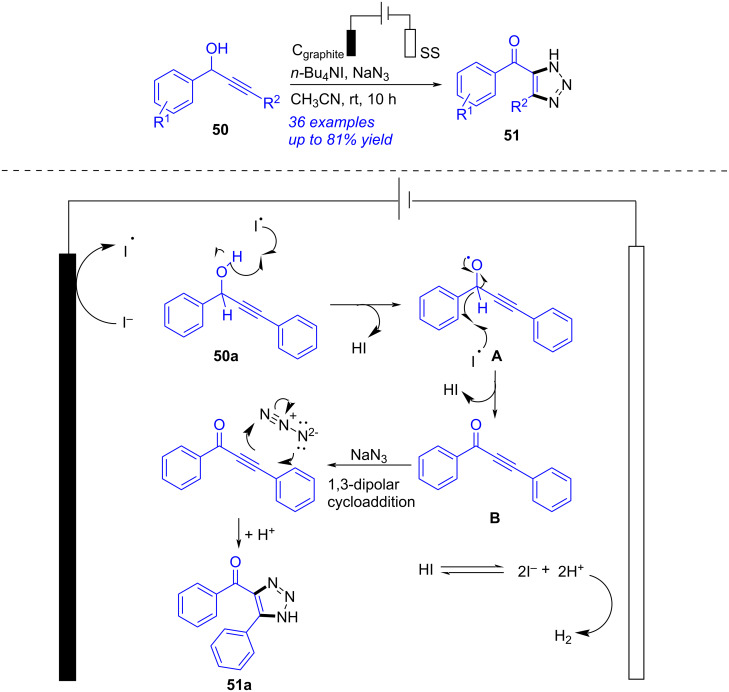
Electrochemical CuAAC to access 1,2,3-triazole.

Early in 2008, an electrochemical copper(I)-catalyzed azide–alkyne cycloaddition (CuAAC) to access 1,2,3-triazole was realized by Finn [272]. But the copper catalyst was still required in that procedure. In 2023, Bera presented an electrochemical oxidative [3 + 2] cycloaddition of secondary propargyl alcohol to access 1,2,3-triazole (Scheme 19) [273]. After probing the reaction systematically, the optimal conditions were presented as following: a mixture of propargyl alcohol **50** (0.7 mmol), NaN_3_ (2.8 mmol), *n-*Bu_4_NI (0.5 mmol) and MeCN (10 mL) under electrolysis (graphite rod as anode, stainless-steel plate as cathode, 11 mA) at rt for 10 h. According to the experimental results and density functional theory (DFT) calculations, a plausible mechanism for this reaction was proposed. Firstly, oxidation of I^−^ at the anode afforded I^•^, which abstracted a hydrogen atom from **50a** to form the intermediate **A** with elimination of HI as a by-product. The second abstraction of a hydrogen atom generated ketone **B**, which then underwent 1,3-dipolar cycloaddition to produce **51a**. This report produced 1,2,3-triazole without any metal catalysts, but the reaction time was relatively long, and the yield remained to enhance.

## Conclusion and Outlook

In conclusion, the construction of organic five-membered rings attracted popular attention due to their distinctive properties and wide applications. Alkynes were extensively used as starting materials or intermediates for the synthesis of five-membered rings. Recently, the electrochemical synthesis of organic five-membered rings from alkynes have been developed due to the superiorities of electrochemical transformations. Indole skeletons were obtained successfully through electrochemical coupling of urea derivatives, dehydrogenative annulation of alkynes with anilines, annulation of *o*-arylalkynylanilines, cyclization of 2-ethynylanilines, selenocyclization of diselenides with 2-ethynylanilines, and enantioselective tandem C–H indolization of 2-alkynylanilines with 3-functionalized indoles. The electrochemical and copper-catalyzed annulation of benzamides and terminal alkynes formed isoindolones in high yields. Isoindolinone could be also afforded via electrochemical 5-*exo-dig* aza-cyclization of 2-alkynylbenzamides and reductive cascade annulation of *o*-alkynylbenzamides. An electrochemical intramolecular 1,2-amino oxygenation of alkynes provided indolizines in reasonable yields. The electrochemical multicomponent reaction was also developed for the construction of oxazole. Pyrrole could be prepared by electrochemical annulation of alkynes with enamides. Electrochemical [3 + 2] cyclization of heteroarylamine was an efficient access towards imidazopyridine. The electrochemical oxidative [3 + 2] cycloaddition of secondary propargyl alcohol produced 1,2,3-triazole. In most of these above reactions, the target cyclic products were obtained in high yields with wide substance scope without any metal catalysts and accessional oxidants.

Although these above electrochemical transformations of alkynes are powerful and green protocols to construct five-membered rings, the development of other reactions to form organic five-membered rings from alkynes and application of the above reported approaches to construct other organic rings are still needed, and the following scientific topics would be focused: (1) the development of a simple, sustainable and electrochemical procedure to synthesize organic rings from alkynes bearing heteroatoms such as ynamides and thioalkynes would be enhanced in future research; (2) since axial chirality is critical in natural products and pharmaceuticals, it would be significant to apply the electrochemical annulation of alkynes in formation of organic rings with axial chirality; (3) to satisfy the requirements of green and sustainable chemistry, it could be necessary to develop the electrochemical transformations of alkynes towards organic rings in aqueous solution, ionic liquid or deep eutectic solvents (DESs) with recycle of solvents and electrolytes.

**Table 1:** About the Authors.

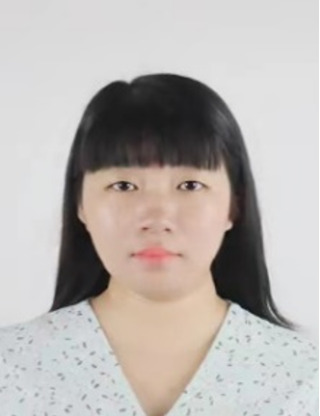	**Lifen Peng** received her Ph.D. under the supervision of Prof. Akihiro Orita and Prof. Junzo Otera at Okayama University of Science, Japan, in 2014. She moved to The Chinese University of Hong Kong and joined Prof. Henry N. C. Wong’s group in Oct. 2014. She became a lecturer at Hunan University of Science and Technology in 2015, and was promoted to an associate professor in 2020. Her research interest is transition metal catalysis, synthesis of alkynes and heterocycles.
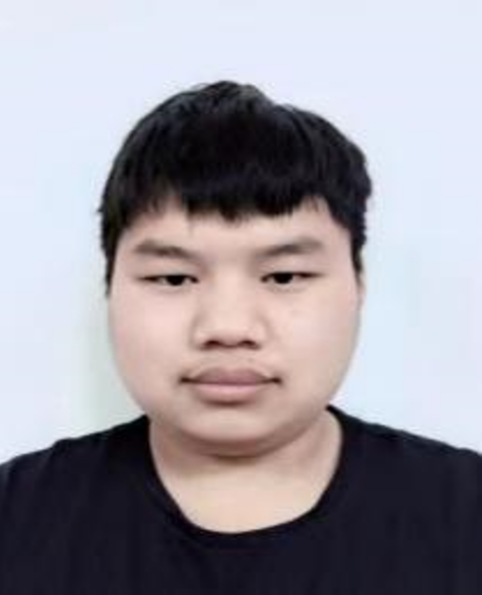	**Ting Wang** received his BS degree at Huaihua University in June 2023. He joined Prof. Lifen Peng’s group and did Master's research at Hunan University of Science and Technology in September 2023. Currently, he is a postgraduate student at Hunan University of Science and Technology majoring in chemical. His research interests mainly focused on transition-metal catalysis, synthesis of alkynes and heterocycles.
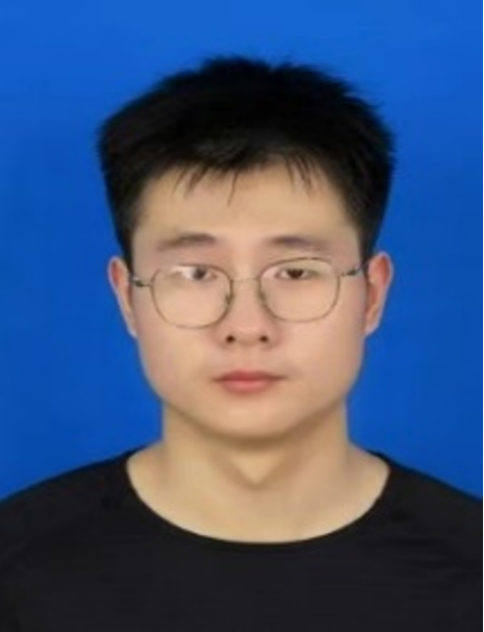	**Zhiwen Yuan** is an undergraduate student at College of Chemistry and Chemical Engineering, Hunan University of Science and Technology, majoring in applied chemistry. His hometown is Ji’an city Jiangxi province. He took part in Prof. Lifen Peng’s group in 2022. Now, he did organic experiments after the class. His current research interest is organic reactions, including transition metal catalysis, the coupling reaction of alkyne, synthesis of cyclic alkynes and heterocycles.
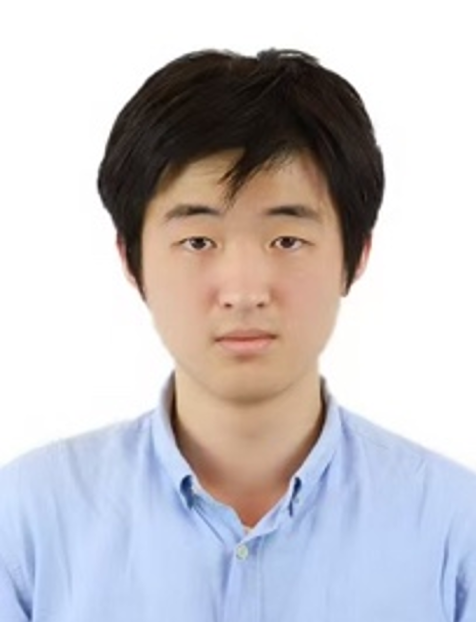	**Bin Li** is an undergraduate student majoring in chemistry at the School of Science and Engineering, The Chinese University of Hong Kong, Shenzhen. He joined Prof. Henry N. C. Wong and Prof. Xiaoshui Peng’s group in 2023, in which he conducted research on organic synthesis of natural and non-natural molecules. His research interests are focusing on syntheses of functionally important organic molecules.
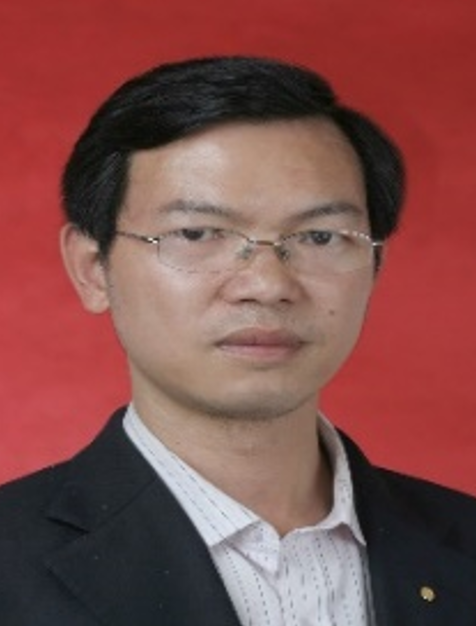	**Zilong Tang** received his Ph.D. under the supervision of Prof. Leon Ghosez at University of Louvain, Belgium in 2004. He joined Prof. Myrargue’s group as a postdoctor at University of Paris XI in France. He is an executive director of the Hunan Chemical Society, a chairman of Fine Chemical Professional Committee of Hunan Chemical Society, a high-level talent in Xiangtan and excellent supervisor of HUST. His interest is pharmacochemistry and organic synthesis.
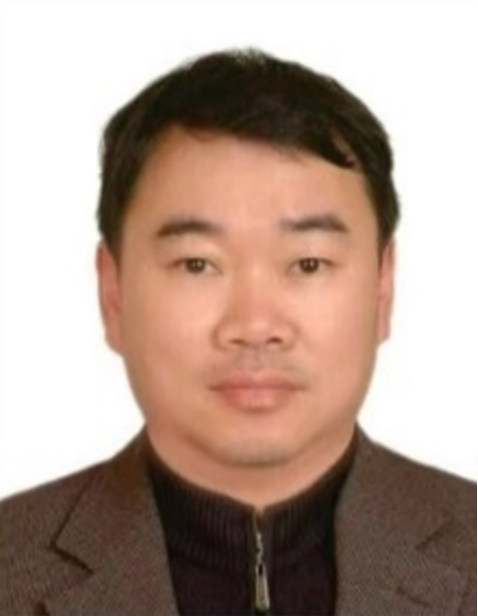	**Xirong Liu** is a chairman of Hunan Norchem Pharmaceutical Co., Ltd. Currently, he is a Ph.D. student at College of Chemistry and Chemical Engineering, Hunan University, doing his thesis under the supervision of Professor Guofang Jiang. He obtained a M. Sc. degree from West China Medical University. His current research interests focus on the enzyme-catalyzed synthesis of steroids, and bioactive natural products.
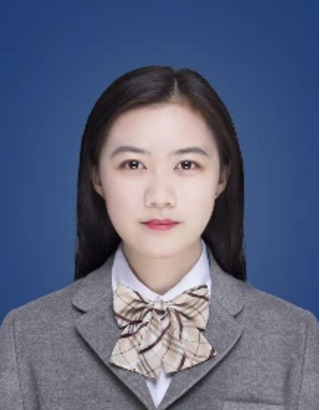	**Hui Li** received her BS degree at Hunan University of Science and Technology in June 2022. She did Master's research at Hunan University in September 2022. She received her Master's degree majoring in chemical engineering under the supervision of Prof. Xinhua Xu at Hunan University in 2025. Her research interests mainly focused on transition-metal catalysis, green organic reactions and the synthesis of natural products.
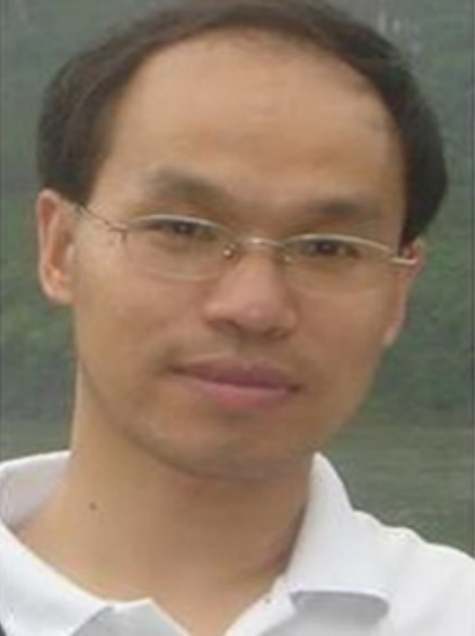	**Guofang Jiang** obtained his Ph.D. from Shanghai Institute of Organic Chemistry, Chinese Academy of Sciences in 1999. Then he moved to Hunan University. From 2002 to 2004, he did his postdoctoral research fellowship at Kyoto University. After that, he returned to Hunan University. He is now a professor and doctoral supervisor at Hunan University. His research is focused on organic synthesis, fine chemicals, biomimetic catalysis, functional materials, electrocatalytic oxidation treatment for wastewater.
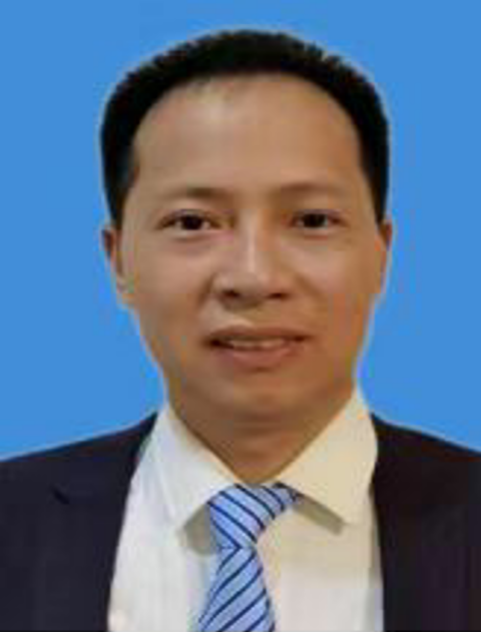	**Chunling Zeng** received his Ph.D. under the supervision of Prof. Xinhua Xu at Hunan University in 2023. He obtained a B. Sc. degree from Hunan University of Chinese Medicine, and his M. Sc. degree from Sichuan University. He spends 15 years as a researcher (2010–now) at Hunan Norchem Pharmaceutical Co., Ltd. His current research interests focus on the enzyme-catalyzed synthesis of steroids and green synthetic chemistry.
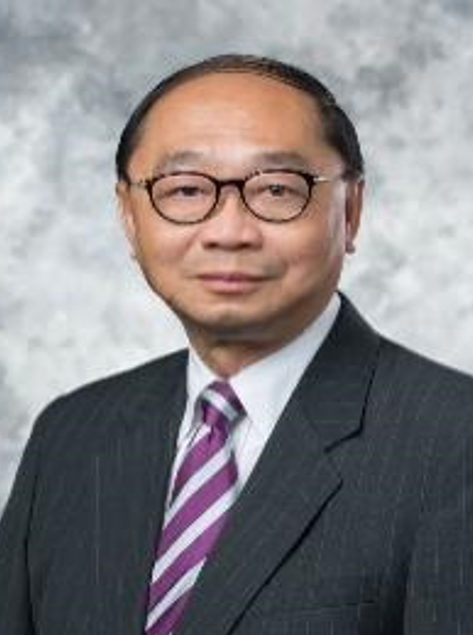	**Henry N. C. Wong** was born in Hong Kong and obtained his B.Sc. degree from The Chinese University of Hong Kong and his Ph.D. degree from University College London (with Prof. Franz Sondheimer) in 1976. After two years at Harvard University as a postdoctoral associate with Prof. Robert B. Woodward, he returned to University College London as a Ramsay Memorial Fellow. From 1980 to 1982, Wong did research at the Shanghai Institute of Organic Chemistry, the Chinese Academy of Sciences. In 1982, he returned to Hong Kong and is now an Emeritus Professor of Chemistry and a Research Professor therein. Concurrently, he is also X. Q. Deng Presidential Chair Professor at The Chinese University of Hong Kong (Shenzhen). His research interests are concerned with syntheses of natural and non-natural molecules as well as synthetic methodologies.
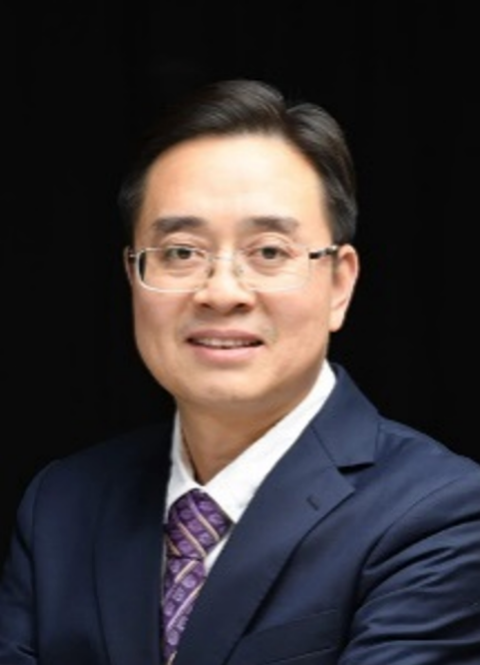	**Xiao-Shui Peng** received his B.Sc. and M.Sc. degrees from Lanzhou University in 1999 and 2002, respectively, under the guidance of Prof. Xin-Fu Pan. In 2006, he obtained his Ph.D. from The Chinese University of Hong Kong, where he worked on the total synthesis of pallavicinin under the supervision of Prof. Henry Wong. After completing his postdoctoral research fellowship with Prof. K. C. Nicolaou and Prof. David Y. K. Chen on the cortistatins project at CSL@Biopolis, Singapore, he returned to The Chinese University of Hong Kong in 2009 as a Research Assistant Professor and then was a Research Associate Professor until 2020. He is now an Associate Professor at The Chinese University of Hong Kong (Shenzhen). His research is focused on the development of novel “bioinspired” strategies and methodologies for the total synthesis of structurally complex and biologically significant natural products.

## Data Availability

Data sharing is not applicable as no new data was generated or analyzed in this study.
